# Social Determinants of Health and 30-Day Mortality After Inpatient Elective Surgery

**DOI:** 10.1001/jamanetworkopen.2025.53228

**Published:** 2026-01-12

**Authors:** Ashwin Sankar, Josephine Ding, Benjamin Black, Andrew S. Wilton, Stephen W. Hwang, Duminda N. Wijeysundera, Nancy N. Baxter, David Gomez

**Affiliations:** 1 Li Ka Shing Knowledge Institute, St Michael’s Hospital, Toronto, Ontario, Canada; 2 Department of Anesthesia, Unity Health Toronto–St Michael’s Hospital, Toronto, Ontario, Canada; 3 ICES Central, Toronto, Ontario, Canada; 4 Department of Anesthesiology and Pain Medicine, University of Toronto, Toronto, Ontario, Canada; 5 Temerty Faculty of Medicine, University of Toronto, Toronto, Ontario, Canada; 6 Division of General Surgery, Unity Health Toronto–St Michael’s Hospital, Toronto, Ontario, Canada; 7 Institute of Health Policy, Management and Evaluation, University of Toronto, Toronto, Ontario, Canada; 8 MAP Centre for Urban Health Solutions, St Michael’s Hospital, Unity Health Toronto, Toronto, Ontario, Canada; 9 Division of General Internal Medicine, Department of Medicine, University of Toronto, Toronto, Ontario, Canada; 10 Faculty of Medicine and Health, University of Sydney, Sydney, New South Wales, Australia; 11 Department of Surgery, Temerty Faculty of Medicine, University of Toronto, Toronto, Ontario, Canada

## Abstract

**Importance:**

Prior research conducted in private for-profit health care systems has suggested that social determinants of health (SDOH) play a role in adverse postoperative outcomes. Whether these findings translate to universal health care systems is unknown.

**Objective:**

To quantify the association of SDOH with risk of 30-day mortality after scheduled inpatient surgery.

**Design, Setting, and Participants:**

This cohort study was conducted in Ontario, Canada’s most populous province. The cohort included consecutive adult patients (aged ≥18 years) who were eligible for Ontario’s universal health insurance and underwent a scheduled inpatient surgical procedure between January 1, 2017, and December 31, 2023. Diagnostic and procedural, demographic, vital statistics, and other data were obtained from linked health administrative databases.

**Exposures:**

SDOH including neighborhood income, immigration status, and migration recency.

**Main Outcome and Measure:**

Death within 30 days of index surgery. Logistic regression models were used to estimate the adjusted and unadjusted odds ratios (AORs and ORs) of the association of each SDOH with 30-day mortality.

**Results:**

Overall, 1 036 759 patients (median [IQR] age, 66 [56-74] years; 526 158 females [50.8%]) who underwent a range of scheduled inpatient surgical procedures were included. Of these patients, 1780 (0.9%) from the lowest-income areas died, as did 1307 (0.6%) from the highest-income areas. Patients from the lowest-income areas were at 52.0% increased odds of death (OR, 1.52; 95% CI, 1.42-1.64) compared with those from the highest-income areas. This association persisted with models partially adjusted for demographic and procedural factors (AOR, 1.54; 95% CI, 1.44-1.66) and fully adjusted for comorbidities (AOR, 1.43; 95% CI, 1.33-1.54). A dose-response association was demonstrated between neighborhood income and mortality, with odds of death increasing with diminishing income (eg, quintile 3 vs quintile 5: AOR, 1.18 [95% CI, 1.10-1.27]; quintile 2 vs quintile 5: AOR, 1.32 [95% CI, 1.22-1.42]). There was evidence of effect modification of the association between neighborhood income and mortality by procedure complexity (eg, effect estimate for quintile 4 and high complexity: –0.0776 [95% CI, –0.2722 to 0.1169]; *P* = .002). Immigrant and refugee status and recent migration (<5 years) demonstrated reduced odds of mortality in unadjusted analyses, but these associations diminished with risk adjustment.

**Conclusions and Relevance:**

In this cohort study, residency in lowest-income neighborhood was associated with increased risk of postoperative mortality despite adjustment for patient, procedure, and hospital factors. Improving postoperative outcomes likely requires addressing underlying SDOH disparities.

## Key Points

**Question:**

How are social determinants of health (neighborhood income, immigration status, and migration recency) associated with 30-day mortality after inpatient elective surgery in a universal health care system?

**Findings:**

In this cohort study including 1 036 759 patients who underwent scheduled surgical procedures, patients from the lowest-income neighborhoods had higher odds of 30-day mortality than those from the highest-income areas, even after adjusting for patient, procedure, and hospital factors. The association showed a dose-response pattern and persisted across study periods; immigration-related factors were not associated with mortality.

**Meaning:**

In this study, lower neighborhood income was associated with higher postoperative mortality, suggesting that improving surgical outcomes may require addressing disparities in social determinants of health.

## Teaser

This cohort study examines the association of neighborhood-level income, immigration status, and migration recency with 30-day mortality among adults in Ontario, Canada, who underwent inpatient elective surgical procedures between 2017 and 2023.

## Introduction

Social determinants of health (SDOH)—the conditions in which people are born, grow, work, live, and age—account for 30% to 55% of health outcomes.^1^ Despite advances in perioperative medicine, nearly 2% of patients die within 30 days of surgery.^2,3,4^ This number has not changed within the past decade,^2^ suggesting that broader systemic factors, including SDOH, are associated with outcomes of surgery.

Although Canada has a universal health care system, where cost is in theory not a barrier to access surgical care, research suggests that SDOH modify access to and outcomes of certain surgical procedures.^5,6^ For example, individuals from low-income neighborhoods experience longer hospital stays and higher rates of non–home discharge after inpatient scheduled surgery compared with individuals from high-income neighborhoods.^7^ During the COVID-19 pandemic, immigration status was associated with rates of scheduled surgery.^8^ The role of these factors in mortality after a broad spectrum of surgical procedures remains unknown in the Canadian context. To address this knowledge gap, we conducted a population-based cohort study in Ontario, Canada, aiming to quantify the association of SDOH with risk of 30-day mortality after scheduled inpatient surgery between 2017 and 2023.

## Methods

We conducted a population-based retrospective cohort study of all adults (aged ≥18 years) living in Ontario, Canada, who were eligible for the Ontario Health Insurance Plan (OHIP) and underwent a scheduled inpatient surgical procedure between January 1, 2017, and December 31, 2023, using linked health administrative databases. Section 45 of Ontario’s Personal Health Information Protection Act authorizes the use of data in this study and does not require review by a Research Ethics Board or informed consent. We followed the Reporting of Studies Conducted Using Observational Routinely Collected Data (RECORD) reporting guideline.

### Data Sources

Almost all Ontario residents receive access to publicly funded universal health care through OHIP (exceptions are military personnel, who receive federal funding, and temporary visitors to the province). Health care data in Ontario are recorded in registries that are used for administration of the health care system. ICES (formerly known as the Institute for Clinical Evaluative Sciences) is an independent, nonprofit research institute that houses health administrative data in Ontario and whose legal status under Ontario’s health information privacy law allows it to collect and analyze health care and demographic data, without patient consent, for the purpose of health system evaluation and improvement.^9^ We used several databases linked on an individual level: the Canadian Institute for Health Information Discharge Abstract Database (CIHI-DAD), which captures diagnostic and procedural data for all hospital admissions; the OHIP dataset, which captures physician claims; the Registered Persons Database, which captures vital statistics (eg, births, deaths) and demographic data on all OHIP-eligible residents; the National Ambulatory Care Reporting System, which captures emergency care utilization; the Continuing Care Reporting System, which captures long-term and respite care utilization; and the Immigration, Refugees and Citizenship Canada Permanent Resident database, which captures demographic information on permanent Canadian residents from 1985, including immigration class (economic or family immigrant or refugee) and duration of residency in Canada. These datasets were linked using unique encoded identifiers and analyzed at ICES.

### Selected Surgical Procedures

We identified all therapeutic procedures occurring between January 1, 2017, and December 31, 2023, using section 1 (eg, physical or physiological therapeutic interventions) of the Canadian Classification of Health Interventions, which captures the type of procedure, setting, and the body system where it was performed.^10^ We then limited our dataset to scheduled inpatient procedures (CIHI-DAD record with scheduled admission type) conducted in the operating room (based on procedure location). We excluded obstetrical interventions and procedures conducted in the interventional radiology suite, cardiac catheterization laboratory, emergency department, and endoscopy unit or on hospital wards. We made the a priori determination (ie, without study outcome data) to exclude patients who underwent low-risk procedure types that are generally conducted as outpatient surgery. These procedures, such as eye and skin surgery, have negligible surgical risk of mortality.

### Exposures

We operationalized SDOH as neighborhood income as well as immigration status and migration recency. Income was defined based on the material resources dimension of the Ontario Marginalization Index, an empirically validated, area-based index derived from Census data designed to facilitate analyses of health inequities across several distinct dimensions of marginalization (eg, income, quality of housing, educational level, and family structure).^11^ Neighborhood income was operationalized as a 5-level ordinal variable, with the least marginalized quintile (ie, quintile 5, highest income area) serving as the reference group. Immigration status was based on immigration visa class as either immigrant or refugee.^12^ Migration recency (ie, duration of residence in Canada) was classified as 0 to 5 years, 6 to 10 years, and Canadian-born or long-term resident, because Statistics Canada often uses 5 years in its definition of recent or new immigrant populations in Canada.

### Outcomes

The primary outcome was death within 30 days of index surgery. Thirty-day mortality is a well-established end point in perioperative medicine that reflects the early postoperative course and is the most widely described follow-up duration after scheduled surgery.^13,14,15^

### Covariates

We adjusted for variables that might influence the association between SDOH and mortality. We identified demographic characteristics (age and sex) from the Registered Persons Database and extracted the procedure site from the CIHI-DAD, which has high accuracy. We used the Operative Stress Score (OSS) to classify procedures into low (OSS 1-2; eg, laparoscopic ovarian cystectomy), medium (OSS 3; eg, mastectomy), or high complexity (OSS 4-5; eg, total esophagectomy).^16^ These OSSs were assigned by a surgeon (B.B.) and an anesthesiologist (J.D.) independently, and their disagreements were resolved by review and consensus between a consultant surgeon (D.G.) and consultant anesthesiologist (A.S.). Rural status was assigned to patients based on the Rurality Index for Ontario, a validated measure based on Census dissemination area representing urban (0-9), semiurban (10-30), and remote rural areas (≥40).^17^ We also considered the teaching status of the institution and year of index surgery.

Patient comorbidities that may confound the exposure and outcome associations were considered. Validated algorithms were used to determine whether patients had heart failure, hypertension, and/or diabetes. The *International Statistical Classification of Diseases and Related Health Problems, Tenth Revision Canada* diagnostic codes in the CIHI-DAD before index surgery were used to characterize other comorbidities, including coronary artery disease, atrial fibrillation, cerebrovascular disease, any cancer, peripheral vascular disease, chronic kidney disease, and liver disease.^18,19,20,21,22,23^ The Johns Hopkins ACG System, version 10, was used to generate clinically oriented categories of comorbidity burden by summing aggregated diagnosis groups. Comorbidity burden was operationalized as low (scores 0-5), medium (5-9), or high (≥10), as previously described.^24,25^

### Statistical Analysis

We described patient characteristics over the study period (2017-2023) and compared patients who underwent surgery prior to (2017-2019) vs after (2020-2023) 2020 using standardized differences. Standardized differences of less than 10% represent negligible differences. We described the incidence of death within 30 days of index surgery by neighborhood income and immigration status.

Adjusted and unadjusted odds ratios (AORs and ORs) separately evaluating the association of each exposure with 30-day mortality were computed using logistic regression models. Given that comorbidities may potentially attenuate the association between SDOH and 30-day mortality, models were generated with partial OR adjustment (adjusted for age, sex, rural status, procedure type and complexity, teaching hospital status, and year) and full OR adjustment (additionally adjusted for comorbidities and comorbidity burden based on the ACG system).

We generated models over the study period and separately for patients who underwent surgery before or after 2020 to explore whether mortality after surgery changed following the COVID-19 pandemic beginning in 2020. We assessed effect modification by period (2017-2019 vs 2020-2023) by including an interaction term of each exposure with period and by comparing models with vs without the interaction term using a likelihood ratio test.

We conducted 2 sensitivity analyses, both of which were exploratory. First, we evaluated effect modification by procedure complexity by including an interaction term between procedure complexity and each SDOH with the primary outcome and by comparing models with vs without the interaction term using a likelihood ratio test. Second, we accounted for clustering within hospitals by evaluating the association of exposures with mortality using a multilevel generalized linear mixed model. Hospitals were modeled with random intercepts, and the only hospital-level covariate was teaching status. We evaluated whether the effect estimates for exposures varied in multilevel models and generated the median OR (which estimates the median increase in odds of the outcome when moving from a lower-risk to a higher-risk hospital with otherwise identical covariates) and variance partition coefficient (which estimates the proportion of explained variation attributed to hospital factors). We also conducted an exploratory analysis of time to death, and proportion of in-hospital vs out-of-hospital death, by income quintile and compared quintiles using analysis of variance and χ^2^ test, respectively.

All analyses were conducted from February 2024 to September 2025 using SAS, version 9.4 (SAS Institute). Two-tailed *P* < .05 was considered statistically significant.

## Results

A total of 1 036 759 patients (median [IQR] age, 66 [56-74] years; 526 158 females [50.8%], 510 601 males [49.2%]) who underwent a range of scheduled inpatient surgical procedures were included (Table 1). Of these patients, 49.3% (511 273) had surgery after 2020.

**Table 1.** Patient Characteristics

Characteristic	Patients, No. (%)			Standardized difference
	Overall (N = 1 036 759)	2017-2019 (n = 525 486)	2020-2023 (n = 511 273)	
Age, median (IQR), y	66 (56-74)	65 (55-74)	67 (57-74)	0.073
Sex				
Female	526 158 (50.8)	268 473 (51.1)	257 685 (50.4)	0.014
Male	510 601 (49.2)	257 013 (48.9)	253 588 (49.6)	
Year of index surgery				
2017	164 645 (15.9)	164 645 (31.3)	0	NA
2018	166 299 (16.0)	166 299 (31.6)	0	NA
2019	165 724 (16.0)	165 724 (31.5)	0	NA
2020	130 523 (12.6)	28 818 (5.5)	101 705 (19.9)	NA
2021	132 308 (12.8)	0	132 308 (25.9)	NA
2022	131 505 (12.7)	0	131 505 (25.7)	NA
2023	145 755 (14.1)	0	145 755 (28.5)	NA
Neighborhood income				
Quintile 1 (lowest)	214 973 (20.7)	110 026 (20.9)	104 947 (20.5)	0.010
Quintile 2	220 948 (21.3)	110 176 (21.0)	110 772 (21.7)	0.017
Quintile 3	209 542 (20.2)	104 611 (19.9)	104 931 (20.5)	0.015
Quintile 4	194 910 (18.8)	99 685 (19.0)	95 225 (18.6)	0.009
Quintile 5 (highest)	188 101 (18.1)	96 616 (18.4)	91 485 (17.9)	0.013
Rural status				
Urban	654 054 (63.1)	332 789 (63.3)	321 265 (62.8)	0.010
Semi-urban	263 862 (25.5)	132 878 (25.3)	130 984 (25.6)	0.008
Remote rural	107 177 (10.3)	53 947 (10.3)	53 230 (10.4)	0.005
Immigration status				
Immigrant	17 526 (1.7)	9931 (1.9)	7595 (1.5)	0.031
Canadian-born or long-term resident	1 014 898 (97.9)	513 141 (97.7)	501 757 (98.1)	0.034
Refugee	4335 (0.4)	2414 (0.5)	1921 (0.4)	0.013
Migration recency				
0-5 y	9899 (1.0)	6071 (1.2)	3828 (0.7)	0.042
6-10 y	11 962 (1.2)	6274 (1.2)	5688 (1.1)	0.008
Canadian-born or long-term resident	1 014 898 (97.9)	513 141 (97.7)	501 757 (98.1)	0.034
Comorbidities				
Heart failure	91 431 (8.8)	44 847 (8.5)	46 584 (9.1)	0.020
Diabetes	279 423 (27.0)	140 032 (26.6)	139 391 (27.3)	0.014
Hypertension	633 550 (61.1)	321 641 (61.2)	311 909 (61.0)	0.004
Cancer	346 304 (33.4)	166 711 (31.7)	179 593 (35.1)	0.072
Myocardial infarction	71 007 (6.8)	35 406 (6.7)	35 601 (7.0)	0.009
CAD	48 756 (4.7)	24 795 (4.7)	23 961 (4.7)	0.002
Atrial fibrillation	32 237 (3.1)	15 708 (3.0)	16 529 (3.2)	0.014
Stroke or TIA	16 232 (1.6)	7872 (1.5)	8360 (1.6)	0.011
CKD	7716 (0.7)	4003 (0.8)	3713 (0.7)	0.004
Liver disease	5241 (0.5)	2354 (0.4)	2887 (0.6)	0.016
Comorbidity burden				
Low	123 619 (11.9)	60 346 (11.5)	63 273 (12.4)	0.028
Medium	365 324 (35.2)	184 949 (35.2)	180 375 (35.3)	0.002
High	547 816 (52.8)	280 191 (53.3)	267 625 (52.3)	0.020
Site of surgery				
Nervous system	21 220 (2.0)	9486 (1.8)	11 734 (2.3)	0.035
Respiratory	41 165 (4.0)	19 406 (3.7)	21 759 (4.3)	0.029
Cardiovascular	141 318 (13.6)	68 532 (13.0)	72 786 (14.2)	0.035
Gastrointestinal	150 773 (14.5)	70 738 (13.5)	80 035 (15.7)	0.062
Urological	128 435 (12.4)	66 631 (12.7)	61 804 (12.1)	0.018
Gynecological	112 558 (10.9)	60 221 (11.5)	52 337 (10.2)	0.039
Musculoskeletal	441 290 (42.6)	230 472 (43.9)	210 818 (41.2)	0.053
Teaching hospital	479 516 (46.3)	241 282 (45.9)	238 234 (46.6)	0.014
Procedure complexity				
Low	458 050 (44.2)	243 393 (46.3)	214 657 (42.0)	0.087
Medium	335 703 (32.4)	166 749 (31.7)	168 954 (33.0)	0.028
High	243 006 (23.4)	115 344 (21.9)	127 662 (25.0)	0.071

Abbreviations: CAD, coronary artery disease; CKD, chronic kidney disease; NA, not applicable; TIA, transient ischemic attack.

Patients who underwent surgery in the 2017 to 2019 vs the 2020 to 2023 periods were similar in terms of age, sex, individual comorbidities, and overall comorbidity burden. Procedures were primarily conducted in urban areas among Canadian-born or long-term residents. A range of procedures was conducted, with similar distribution of surgery system and procedures conducted in teaching hospitals across both periods (Table 1).

The incidence of 30-day mortality by neighborhood income, immigration status, and procedural factors are presented in Table 2. Overall, 1780 individuals (0.9%) from the lowest-income areas (quintile 1) died, as did 1307 individuals (0.6%) from the highest-income areas (quintile 5). Of those who underwent gynecological procedures, 113 (0.1%) died, as did 2767 (2.0%) of those who underwent cardiovascular procedures.

**Table 2.** Frequency of 30-Day Mortality by Social Determinants of Health and Surgical Factors

Variable	Patients, No. (%)			Standardized difference
	Overall (N = 1 036 759)	2017-2019 (n = 525 486)	2020-2023 (n = 511 273)	
Neighborhood income				
Quintile 1 (lowest)	1780 (0.9)	808 (0.8)	972 (1.1)	0.023
Quintile 2	1701 (0.9)	765 (0.8)	936 (1.0)	0.023
Quintile 3	1591 (0.8)	717 (0.7)	874 (0.8)	0.017
Quintile 4	1614 (0.7)	741 (0.7)	873 (0.8)	0.014
Quintile 5 (highest)	1307 (0.6)	582 (0.5)	725 (0.7)	0.021
Immigration status				
Canadian-born or long-term resident	7864 (0.8)	3548 (0.7)	4316 (0.9)	0.019
Immigrant	109 (0.6)	56 (0.6)	53 (0.7)	0.017
Refugee	20 (0.5)	9 (0.4)	11 (0.6)	0.029
Migration recency				
Canadian-born or long-term resident	7864 (0.8)	3548 (0.7)	4316 (0.9)	0.019
0-5 y	55 (0.6)	31 (0.5)	24 (0.6)	0.015
6-10 y	74 (0.6)	34 (0.5)	40 (0.7)	0.020
Site of surgery				
Nervous system	304 (1.4)	122 (1.3)	182 (1.6)	0.022
Respiratory	644 (1.6)	327 (1.7)	317 (1.5)	0.019
Cardiovascular	2767 (2.0)	1165 (1.7)	1602 (2.2)	0.036
Gastrointestinal	2161 (1.4)	993 (1.4)	1168 (1.5)	0.005
Urological	799 (0.6)	385 (0.6)	414 (0.7)	0.012
Gynecological	113 (0.1)	56 (0.1)	57 (0.1)	0.005
Musculoskeletal	1205 (0.3)	565 (0.2)	640 (0.3)	0.011
Procedure complexity				
Low	1762 (0.4)	811 (0.3)	951 (0.4)	0.018
Medium	3015 (0.9)	1334 (0.8)	1681 (1.0)	0.021
High	3216 (1.3)	1468 (1.3)	1748 (1.4)	0.008

In unadjusted analyses, patients from the lowest-income areas were at 52.0% increased odds of death (OR, 1.52; 95% CI, 1.42-1.64) compared with those from the highest-income areas (Table 3). This association persisted with partial adjustment (AOR, 1.54; 95% CI, 1.44-1.66) and full adjustment (AOR, 1.43; 95% CI, 1.33-1.54). A dose-response association was demonstrated between neighborhood income and mortality, with odds of death increasing across quintiles of diminishing income (eg, quintile 3 vs quintile 5: AOR, 1.18 [95% CI, 1.10-1.27]; quintile 2 vs quintile 5: AOR, 1.32 [95% CI, 1.22-1.42]). While immigrant and refugee status and recent migration (<5 years) were associated with reduced odds of mortality in unadjusted analyses, these associations diminished with risk adjustment (Table 3). In fully adjusted models, immigration status was associated with an increased likelihood of mortality (AOR, 1.33; 95% CI, 1.10-1.61) for the entire study period, suggesting that reduced comorbidity may explain the diminished risk of mortality in unadjusted analyses.

**Table 3.** Associations Between Social Determinants of Health and 30-Day Mortality

Variable	2017-2023			2017-2019			2020-2023		
	OR (95% CI)	AOR (95% CI), partial adjustment	AOR (95% CI), full adjustment	OR (95% CI)	AOR (95% CI), partial adjustment	AOR (95% CI), full adjustment	OR (95% CI)	AOR (95% CI), partial adjustment	AOR (95% CI), full adjustment
Neighborhood income									
Quintile 1 (lowest)	1.52 (1.42-1.64)	1.54 (1.44-1.66)	1.43 (1.33-1.54)	1.54 (1.39-1.72)	1.54 (1.38-1.71)	1.42 (1.27-1.59)	1.51 (1.37-1.66)	1.55 (1.40-1.71)	1.44 (1.30-1.59)
Quintile 2	1.42 (1.32-1.52)	1.38 (1.28-1.49)	1.32 (1.22-1.42)	1.43 (1.28-1.59)	1.37 (1.23-1.53)	1.30 (1.17-1.45)	1.41 (1.27-1.55)	1.39 (1.26-1.53)	1.33 (1.20-1.47)
Quintile 3	1.23 (1.15-1.33)	1.22 (1.13-1.31)	1.18 (1.10-1.27)	1.28 (1.15-1.43)	1.25 (1.12-1.39)	1.21 (1.08-1.35)	1.19 (1.08-1.32)	1.19 (1.08-1.32)	1.15 (1.04-1.27)
Quintile 4	1.20 (1.11-1.29)	1.20 (1.11-1.29)	1.17 (1.08-1.25)	1.26 (1.13-1.41)	1.25 (1.12-1.39)	1.22 (1.09-1.36)	1.14 (1.03-1.25)	1.16 (1.04-1.28)	1.12 (1.01-1.24)
Quintile 5 (highest)	1 [Reference]	1 [Reference]	1 [Reference]	1 [Reference]	1 [Reference]	1 [Reference]	1 [Reference]	1 [Reference]	1 [Reference]
Immigration status									
Canadian-born or long-term resident	1 [Reference]	1 [Reference]	1 [Reference]	1 [Reference]	1 [Reference]	1 [Reference]	1 [Reference]	1 [Reference]	1 [Reference]
Immigrant	0.80 (0.66-0.96)	1.11 (0.92-1.34)	1.33 (1.10-1.61)	0.81 (0.62-1.05)	1.14 (0.87-1.49)	1.42 (1.08-1.86)	0.81 (0.61-1.06)	1.10 (0.83-1.44)	1.27 (0.97-1.68)
Refugee	0.58 (0.37-0.89)	0.97 (0.62-1.51)	1.05 (0.68-1.64)	0.52 (0.27-1.01)	0.90 (0.47-1.73)	1.04 (0.54-2.02)	0.65 (0.36-1.18)	1.05 (0.58-1.91)	1.07 (0.59-1.95)
Migration recency									
Canadian-born or long-term resident	1 [Reference]	1 [Reference]	1 [Reference]	1 [Reference]	1 [Reference]	1 [Reference]	1 [Reference]	1 [Reference]	1 [Reference]
0-5 y	0.71 (0.54-0.93)	1.04 (0.80-1.37)	1.32 (1.01-1.73)	0.73 (0.51-1.04)	1.06 (0.74-1.51)	1.41 (0.98-2.02)	0.72 (0.48-1.08)	1.06 (0.70-1.59)	1.25 (0.83-1.88)
6-10 y	0.79 (0.63-0.99)	1.12 (0.89-1.41)	1.25 (0.99-1.58)	0.77 (0.55-1.09)	1.14 (0.81-1.60)	1.30 (0.92-1.83)	0.81 (0.59-1.10)	1.11 (0.81-1.52)	1.22 (0.89-1.68)

Abbreviations: AOR, adjusted odds ratio; OR, odds ratio.

The associations described between neighborhood income and 30-day mortality were consistent in unadjusted, partially adjusted, and fully adjusted analyses both in the 2017 to 2019 and the 2020 to 2023 periods (Table 3). There was no evidence of effect modification by period (likelihood ratio: *P* = .54 for neighborhood income, *P* = .97 for immigration status, and *P* = .99 for migration recency) (eTable 1 in Supplement 1).

Exploratory analyses suggested effect modification by procedure complexity of the association between neighborhood income and mortality (eg, quintile 4 and high complexity: –0.0776 [95% CI, –0.2722 to 0.1169]; *P* = .002) (eTable 2 in Supplement 1). Procedure complexity did not modify associations of immigration status or migration recency with mortality (eTable 2 in Supplement 1). In models that included an interaction between neighborhood income and procedure complexity, we assessed associations of income quintile and mortality, stratified by procedure complexity (Table 4). Among patients with low-complexity procedures, only those from the lowest-income areas had significantly increased odds of death after partial adjustment (AOR, 1.59; 95% CI, 1.37-1.85) and full adjustment (AOR, 1.45; 95% CI, 1.24-1.68) compared with patients from highest-income areas. Among patients with medium- and high-complexity procedures, the odds of mortality increased with diminishing income status.

**Table 4.** Effect Modification by Procedure Complexity of the Associations Between Social Determinants of Health and Mortality

Variable	Low complexity		Medium complexity		High complexity	
	AOR (95% CI), partial adjustment	AOR (95% CI), full adjustment	AOR (95% CI), partial adjustment	AOR (95% CI), full adjustment	AOR (95% CI), partial adjustment	AOR (95% CI), full adjustment
Neighborhood income						
Quintile 1 (lowest)	1.59 (1.37-1.85)	1.45 (1.24-1.68)	1.57 (1.39-1.78)	1.48 (1.31-1.68)	1.46 (1.30-1.63)	1.35 (1.21-1.52)
Quintile 2	1.14 (0.97-1.34)	1.09 (0.92-1.28)	1.54 (1.36-1.74)	1.50 (1.33-1.69)	1.37 (1.22-1.53)	1.29 (1.15-1.45)
Quintile 3	1.18 (1.01-1.38)	1.14 (0.97-1.33)	1.31 (1.16-1.48)	1.29 (1.14-1.46)	1.15 (1.02-1.29)	1.11 (0.99-1.25)
Quintile 4	1.09 (0.93-1.28)	1.06 (0.90-1.24)	1.26 (1.12-1.43)	1.24 (1.09-1.40)	1.19 (1.06-1.34)	1.16 (1.03-1.30)
Quintile 5 (highest)	1 [Reference]	1 [Reference]	1 [Reference]	1 [Reference]	1 [Reference]	1 [Reference]
Immigration status						
Canadian-born or long-term resident	1 [Reference]	1 [Reference]	1 [Reference]	1 [Reference]	1 [Reference]	1 [Reference]
Immigrant	1.26 (0.87-1.83)	1.57 (1.08-2.29)	1.14 (0.80-1.61)	1.44 (1.02-2.05)	1.03 (0.77-1.38)	1.17 (0.87-1.57)
Refugee	1.21 (0.57-2.58)	1.31 (0.61-2.82)	0.60 (0.23-1.62)	0.68 (0.25-1.84)	1.06 (0.55-2.06)	1.15 (0.59-2.22)
Migration recency						
Canadian-born or long-term resident	1 [Reference]	1 [Reference]	1 [Reference]	1 [Reference]	1 [Reference]	1 [Reference]
0-5 y	1.16 (0.69-1.95)	1.51 (0.89-2.55)	1.08 (0.66-1.74)	1.43 (0.88-2.33)	0.94 (0.62-1.43)	1.12 (0.74-1.70)
6-10 y	1.32 (0.85-2.04)	1.52 (0.97-2.36)	1.00 (0.64-1.57)	1.19 (0.76-1.85)	1.11 (0.79-1.57)	1.20 (0.85-1.70)

Abbreviation: AOR, adjusted odds ratio.

Multilevel models (Table 5) revealed that after accounting for clustering at the hospital level, patients in the lowest-income neighborhoods had an increased odds of mortality compared with those in the highest-income areas (AOR, 1.40; 95% CI, 1.30-1.51). The magnitude of association between neighborhood income and mortality increased with diminishing income. The median OR in models considering clustering was 1.84 (95% CI, 1.83-1.85), and the variance partition coefficient was 0.11, suggesting that 11% of variance was explained by hospital-level factors. Exploratory analyses of time to death and proportion of in-hospital vs out-of-hospital death revealed no differences by income quintile (eTable 3 in Supplement 1).

**Table 5.** Analyses Adjusted for Hospital-Level Clustering

Variable	AOR (95% CI)
Neighborhood income	
Quintile 1 (lowest)	1.40 (1.30-1.51)
Quintile 2	1.30 (1.20-1.40)
Quintile 3	1.16 (1.08-1.26)
Quintile 4	1.14 (1.06-1.23)
Quintile 5 (highest)	1 [Reference]
Immigration status	
Canadian-born or long-term resident	1 [Reference]
Immigrant	1.38 (1.14-1.68)
Refugee	1.10 (0.70-1.71)
Migration recency	
Canadian-born or long-term resident	1 [Reference]
0-5 y	1.36 (1.04-1.78)
6-10 y	1.31 (1.04-1.65)

Abbreviation: AOR, adjusted odds ratio.

## Discussion

In this population-based cohort study including more than 1 million patients with elective surgical procedures in Ontario, we found that residency in a low-income neighborhood was associated with increased odds of 30-day mortality following inpatient scheduled surgery, even within the context of a universal health care system. The association between low-income neighborhood and postoperative mortality persisted despite adjustment for demographic factors, known patient comorbidities and health status, hospital-level variables, study period, procedure complexity, and within-hospital clustering. While the associations between neighborhood income and postoperative mortality were replicable, exhibited dose-response associations, and persisted through multiple analyses, immigration status did not demonstrate similar associations with mortality.

Overall, these results highlight the important role of socioeconomic status in surgical outcomes. Most prior studies suggesting this association were conducted in countries with private health care systems,^26,27^ but our research showed that disparities in surgical outcomes exist and persist despite access to surgical care under a universal health care system. Various factors associated with the SDOH, beyond those captured in administrative data, may be associated with the observed results. Individuals living in low-income neighborhoods may face diminished access to primary care,^28^ receive inadequate treatment for chronic disease,^29^ encounter challenges in accessing medications,^30^ and present with more severe surgical disease.^31^ Furthermore, the lack of access to health-promoting conditions and increased exposure to risk factors, including alcohol use and smoking, may add to the burden of chronic disease.^32^ Furthermore, social support may be limited, as might the capacity to pay for out-of-hospital supports (eg, additional physiotherapy, in-home care, medical devices and equipment) that are variably covered by Canadian health care.^33^ Taken together, these factors highlight that neighborhood income likely serves as a marker for broader social disadvantage that ultimately is associated with heightened risk of mortality after surgery.

Our multilevel analyses indicated that hospital-level clustering accounted for a modest proportion of variance in outcomes (variance partition coefficient, 0.11; median OR, 1.84). This finding reinforces the importance of both patient-level and hospital-related factors in disparities. Policy interventions targeting upstream SDOH, in addition to hospital-level practices, may therefore be required to increase equity in surgical outcomes. The consistency of observed disparities prior to and after 2020 further underscores the persistence of income-related inequalities in health outcomes and suggests that health care adaptations made during the COVID-19 pandemic in 2020, such as surgical prioritization protocols, did not mitigate underlying socioeconomic inequities.^34,35^

Exploratory analyses suggested that procedure complexity modified the association between neighborhood income and mortality. Among patients who had low-complexity procedures, increased mortality risk was limited to those living in the lowest-income areas, whereas among patients with medium- and high-complexity procedures, mortality risk increased sequentially with decreasing neighborhood income. These results suggest that socioeconomic disadvantage is associated with outcomes across the surgical risk spectrum. As our cohort was limited to individuals who underwent surgery, further research is needed to disentangle the mechanisms underlying these associations, including where in the patient care pathway (access to preoperative optimization, perioperative support, and postdischarge care) that socioeconomically disadvantaged communities require further support.^36,37^ Our exploratory analyses of in-hospital vs out-of-hospital mortality after scheduled surgery demonstrated no differences between the income-quintile groups, suggesting that factors upstream of surgery might require further attention. Future research is needed to examine processes of care in marginalized communities and whether tailored interventions addressing certain processes can mitigate disparities.

This study did not identify an association between immigration status and 30-day mortality, which contrasts with previous literature showing worsened access to scheduled surgery for immigrant populations during the COVID-19 pandemic. Four potential factors may have played a role in these findings. First, immigration rates were low, particularly in 2020 during the first wave of the COVID-19 pandemic.^38^ Second, the healthy immigrant effect, a phenomenon in which recent immigrants have improved overall health due to self-selection and immigration policies favoring healthy individuals,^39^ may be protective in the perioperative context. It may have played a role in the diminished risk of death we observed in unadjusted analyses. Third, it is possible that barriers to accessing surgical care may have selectively reduced the number of immigrants who underwent higher-risk procedures. Fourth, in some immigrant communities, the nature of family structures, such as multigenerational living, may provide greater social support to facilitate early discharge from hospital and aid in postoperative recovery.^40^

The association between neighborhood income and postoperative outcomes is multifaceted and complex, and elucidating the underlying mechanisms is essential for translating these findings into improved patient care. In primary health care settings, a single-question poverty screening tool has demonstrated nearly 98% sensitivity in identifying individuals living below a low-income threshold.^41^ A growing body of evidence further highlights the role of food insecurity in adverse outcomes following trauma and oncologic surgeries.^42,43^ Whether preoperative identification of such high-risk SDOH enhances risk stratification—and whether it enables perioperative clinicians to connect patients with appropriate medical and social support services—warrants further investigation. Furthermore, access to social supports may be influential in shaping postoperative outcomes. Patients from low-income communities often have fewer formal and informal support systems to assist with recovery, such as help with daily activities, medication management, and transportation to follow-up appointments. Their ability to take time away from work to recover after surgery may be limited. This lack of support may be associated with delays in seeking care for complications, reduced adherence to treatment plans, and increased postoperative risk.^44,45^

### Strengths and Limitations

This study has several strengths. These strengths include the large population-based cohort of consecutively scheduled surgical procedures, use of high-quality linked administrative databases, and robust adjustment for demographic and comorbidity confounders.

However, this study also has certain limitations. First, our income measure was based on neighborhood-level deprivation rather than individual-level income, potentially introducing misclassification bias. Previous work in a universal health care system has suggested that area-level measures of income can be used to describe socioeconomic inequities.^46^ Our inability to capture patient-level SDOH, however, limits the evaluation of their importance to health outcomes. Second, unmeasured factors such as differences in health behaviors (eg, smoking, alcohol use, and drug use), social support, and access to community-based care could have a role in the observed associations but were not captured in our administrative health datasets. Third, there is growing evidence that the different SDOH intersect.^1^ The small number of immigrants in this study precluded analyses considering this possibility. Future work is needed to examine the importance of the intersection of various SDOH, such as income and immigration status, and outcomes of surgery. Fourth, individuals from low-income neighborhoods may face diminished access to primary care and may not accrue diagnosis or treatment of comorbid disease; they may appear to have fewer comorbidities as a result. However, we assigned comorbid disease using validated codes with long lookback windows; we further demonstrated consistent associations of neighborhood income with mortality in both partially adjusted (for demographics and procedure) and fully adjusted (additionally adjusted for comorbidities) models. Fifth, given the breadth of the procedures included, we adjusted for complexity but not for underlying disease severity. Future work is needed to examine the importance of SDOH to severity of surgical disease and postoperative outcomes.

## Conclusions

This cohort study highlighted significant neighborhood income–based disparities in mortality following scheduled inpatient surgery in a universal health care system. Future research is warranted to explore the mechanisms underlying these disparities and to develop strategies such as enhanced perioperative support for socioeconomically disadvantaged patients to improve surgical outcomes. Overall, these findings emphasized the need for ongoing efforts to address neighborhood income–related inequities in surgical care and outcomes.

## Notes

Supplement 1. **eTable 1.** Effect Estimates of Interaction Terms of Primary Exposures by Time Period (2017-2019 versus 2020-2023) **eTable 2.** Effect Estimates of Interaction Terms of Primary Exposures by Procedure Complexity **eTable 3.** Exploratory Analysis of Time to Death, and Proportion of In-hospital vs Out-of-hospital Mortality by Neighborhood Income Quintile

Supplement 2.
Data Sharing Statement
