# Supplementary material for: Social Determinants of Health and 30-Day Mortality After Inpatient Elective Surgery
Source: JAMA Netw Open. 2026 Jan 12;9(1):e2553228. doi: 10.1001/jamanetworkopen.2025.53228 (PMC12797094; doi:10.1001/jamanetworkopen.2025.53228)
Supplement: Supplement 1. — eTable 1. Effect Estimates of Interaction Terms of Primary Exposures by Time Period (2017-2019 versus 2020-2023) eTable 2. Effect Estimates of Interaction Terms of Primary Exposures by Procedure Complexity eTable 3. Exploratory Analysis of Time to Death, and Proportion of In-hospital vs Out-of-hospital Mortality by Neighborhood Income Quintile [file jamanetwopen-e2553228-s001.pdf]

Supplemental Online Content

Sankar A, Ding J, Black B, et al. Social determinants of health and 30-day mortality after inpatient elective surgery. *JAMA Netw Open*. 2026;9(1):e2553228. doi:10.1001/jamanetworkopen.2025.53228

eTable 1. Effect Estimates of Interaction Terms of Primary Exposures by Time Period (2017-2019 versus 2020-2023)

eTable 2. Effect Estimates of Interaction Terms of Primary Exposures by Procedure Complexity

eTable 3. Exploratory Analysis of Time to Death, and Proportion of In-hospital versus Out-of-hospital Mortality by Neighbourhood Income Quintile

This supplemental material has been provided by the authors to give readers additional information about their work.

© 2026 Sankar A et al. *JAMA Network Open*

eTable 1: Effect estimates of interaction terms of primary exposures by time period (2017-2019 versus 2020-2023)

			Estimate	LCL	UCL	Global p-value
**IncomeQuintile*TimePeriod**	Q4	Pre-2020	0.0992	0.2479	-0.0495	0.5385
	Q3	Pre-2020	0.0626	0.2119	-0.0867	
	Q2	Pre-2020	-0.0011	0.1461	-0.1482	
	Q1	Pre-2020	0.0078	0.1536	-0.138	
**Immigrant*TimePeriod**	Immigrants	Pre-2020	0.0169	0.3999	-0.366	0.9672
	Refugees	Pre-2020	-0.1101	0.7798	-1	
**MigrationRecency*TimePeriod**	0-5	Pre-2020	0.0194	0.5602	-0.5213	0.9934
	6-10	Pre-2020	-0.0216	0.4434	-0.4865	

© 2026 Sankar A et al. *JAMA Network Open*

eTable 2: Effect estimates of interaction terms of primary exposures by procedure complexity

			Estimate	LCL	UCL	Global p-value
**IncomeQuintile*Complexity**	Q4	High	-0.0776	-0.2722	0.1169	0.0015
	Q4	Medium	-0.1782	-0.3779	0.0215	
	Q3	High	0.0451	-0.1495	0.2396	
	Q3	Medium	-0.1602	-0.3584	0.038	
	Q2	High	-0.1371	-0.3325	0.0583	
	Q2	Medium	-0.3383	-0.5372	-0.1394	
	Q1	High	0.145	-0.0419	0.3318	
	Q1	Medium	0.0368	-0.1551	0.2287	
**Immigrant*Complexity**	Immigrants	High	0.225	-0.2466	0.6966	0.4629
	Immigrants	Medium	0.2267	-0.2813	0.7346	
	Refugees	High	0.1526	-0.8476	1.1529	
	Refugees	Medium	0.9183	-0.3203	2.157	
**MigrationRecency*Complexity**						
	00-05	High	0.2302	-0.4289	0.8893	0.7002
	00-05	Medium	0.2263	-0.4771	0.9296	
	6-10	High	0.1959	-0.3611	0.7528	
	6-10	Medium	0.4095	-0.2125	1.0315	

© 2026 Sankar A et al. *JAMA Network Open*

eTable 3: Exploratory analysis of time to death, and proportion of in-hospital versus out-of-hospital mortality by neighbourhood income quintile

In-hospital versus out-of-hospital mortality and time to death, by income quintile

**Income Quintile**	**Out of hospital mortality (n, %)**	**In hospital mortality (n, %)**		Mean time to death in days (SD)	Median time to death in days [IQR]	
Overall	2,813 (35.1%)	5,180 (64.9%)	F-statistic 1.18 on 4 df, *p*-value = 0.32	13.17 (8.77)	13 [6–21]	Chi-sq 4.42 on 4 df, *p*-value = 0.35
Q1 (lowest)	597 (33.5%)	1,183 (66.5%)		12.96 (8.76)	12 [5–20]	
Q2	587 (34.5%)	1,114 (65.5%)		13.35 (8.82)	13 [6–21]	
Q3	567 (35.6%)	1,024 (64.4%)		13.22 (8.74)	13 [5–20]	
Q4	590 (36.6%)	1,024 (63.4%)		12.92 (8.73)	12 [5–20]	
Q5 (highest)	472 (36.1%)	835 (63.9%)		13.48 (8.78)	13 [6–21]	

© 2026 Sankar A et al. *JAMA Network Open*
